# Real-world insights into bone marrow carcinomatosis in metastatic breast cancer: a retrospective analysis from two large university breast cancer centers

**DOI:** 10.1007/s00432-026-06546-1

**Published:** 2026-07-23

**Authors:** Nikolas Tauber, Melissa Neubacher, Niklas Amann, Jan P. Cieslik, Irene Esposito, Franziska Fick, Catharina Freier, Verena Friebe, Martina Helbig, Franziska Hemptenmacher, Lisbeth Hilmer, Bernadette Jäger, Kerstin Muras, Anna E. M. Polkaehn, Henriette Princk, Eugen Ruckhäberle, Maximilian Seidl, Tanja Fehm, Achim Rody, Fabian Kohls, Maggie Banys-Paluchowski, Natalia Krawczyk

**Affiliations:** 1 University of Luebeck, Department of Obstetrics and Gynecology, University Hospital Schleswig-Holstein Campus Luebeck, 23562 Luebeck, Germany; 2 Department of Gynecology and Obstetrics, Medical Faculty and University Hospital Duesseldorf, Heinrich Heine University Duesseldorf, 40225 Duesseldorf, Germany; 3 Department of Obstetrics and Gynecology, Comprehensive Cancer Center Erlangen- European Metropolitan Region of Nuremberg, University Hospital Erlangen, 91054 Erlangen, Germany; 4 Institute of Pathology, Medical Faculty and University Hospital Duesseldorf, Heinrich Heine University Duesseldorf, 40225 Duesseldorf, Germany

**Keywords:** Bone marrow carcinomatosis, Bone marrow metastases, Metastatic breast cancer, Pancytopenia

## Abstract

**Purpose:**

Bone marrow carcinomatosis (BMC) is a rare but clinically relevant manifestation of metastatic breast cancer (mBC), characterized by diffuse marrow infiltration and hematologic dysfunction. Evidence on epidemiology, clinical presentation, prognosis, and optimal treatment is limited. We performed a bicentric retrospective real-world analysis of patients diagnosed with BMC, representing the largest reported European cohort to date.

**Methods:**

We retrospectively analyzed 37 patients diagnosed with BMC at two German university breast cancer centers between 2010 and 2023. Clinical characteristics, hematologic abnormalities, metastatic patterns, treatments, and overall survival (OS) were evaluated. Incidence estimates were calculated based on institutional breast cancer and mBC populations. A systematic literature review contextualized the findings.

**Results:**

The estimated incidence of BMC was 2.6% among patients with mBC and 0.5% among all breast cancer cases. Most patients had HR-positive/HER2-negative disease (89%). Hematologic abnormalities were frequent, including anemia (84%), thrombocytopenia (76%), and pancytopenia (57%). Median OS after BMC diagnosis was 12 months (95% CI 6.0–38.0). Patients with bone-only metastases did not reach median OS, whereas those with additional metastatic sites had a median OS of 9 months (95% CI 5.0–12.0). Patients receiving endocrine therapy plus CDK4/6 inhibition showed numerically longer OS (34 months, 95% CI 5.0–NA) compared with chemotherapy alone (12 months, 95% CI 5.0–38.0).

**Conclusions:**

BMC remains a rare but challenging complication of mBC. Endocrine-based targeted therapy may improve outcomes in selected HR-positive/HER2-negative patients. Larger multicenter studies are needed to optimize diagnosis and treatment strategies.

**Supplementary Information:**

The online version contains supplementary material available at 10.1007/s00432-026-06546-1.

## Introduction

With 8.2 million cases diagnosed over the past five years, breast cancer remains the most common malignancy among women worldwide (National Cancer Insitute 2025). Prognosis differs markedly between early breast cancer (eBC) and advanced disease stages. Continuous refinement of personalized treatment strategies has led to substantial improvements in patient prognosis and quality of life (Fernández-Pacheco et al. 2025; Ganster et al. 2025; Hill et al. 2025; Nardin et al. 2020; Tauber et al. 2024, 2025). However, the management of patients with advanced breast cancer remains a challenge in daily clinical practice (Lagendijk et al. 2018; Ren et al. 2024); van Dam et al. (2025). The presence of bone marrow carcinomatosis (BMC) represents a rare but severe complication in the progression of metastatic breast cancer (mBC) with poor prognosis. It is characterized by the diffuse infiltration of malignant cells into the bone marrow, leading to significant hematological abnormalities such as anemia, thrombocytopenia, and leukopenia. These complications not only exacerbate the clinical burden but also pose challenges in delivering systemic therapies. While BMC is most commonly associated with malignancies such as prostate cancer and gastric adenocarcinoma, its occurrence in breast cancer remains underexplored, with very limited data available on its incidence, clinical presentation, and management strategies (Kopp et al. 2011).

The pathophysiology of BMC involves complex mechanisms, including extramedullary hematopoiesis, fibrosis, and intrasinusoidal infiltration of malignant cells. These processes result in the characteristic leucoerythroblastic blood smear, which serves as a key diagnostic indicator. However, this finding alone may lack specificity and sensitivity, necessitating confirmation through bone marrow biopsy in many cases (Kopp et al. 2011). Moreover, the overlap of BMC with other conditions such as myelodysplastic syndromes and treatment indicated bone marrow aplasia or fibrosis further complicates diagnosis.

Current international guidelines, including those from the European Society for Medical Oncology (ESMO), the National Comprehensive Cancer Network (NCCN), or the American Society of Clinical Oncology (ASCO), do not specifically provide detailed treatment recommendations for BMC in breast cancer. Given these limitations and the unanswered questions regarding optimal treatment regimens and the identification of predictive biomarkers for this condition, there is a clear need for larger, multi-center trials to better characterize the clinical course, prognostic factors, and treatment outcomes in BMC patients. In particular, given the increasing number of targeted therapy options for mBC and the fact that most BMC cases have been reported in hormone receptor positive (HR positive) human epidermal growth receptor 2 (HER2) negative breast cancer, the question arises as to whether an endocrine-based therapy should represent a preferred treatment option for these patients.

In this study, we retrospectively analyzed 37 cases of BMC diagnosed at two certified University Breast Cancer Centers (Lübeck and Düsseldorf) in Germany between January 2010 and December 2023. By combining retrospective clinical data with an extensive literature review- including pivotal works by Kopp et al. and Niu et al.- we seek to elucidate the epidemiology, clinical presentation, treatment outcomes, and prognostic factors associated with this rare manifestation of metastatic breast cancer (Kopp et al. 2011; Niu et al. 2023). This work represents the largest real-world analysis of BMC in breast cancer patients conducted in Europe to date.

## Methods

### Study design and data collection

The conception, methodology, analysis, and writing of the paper for this real-world analysis were conducted in accordance with the ESMO guidance for reporting oncology real-world evidence (GROW) (Castelo-Branco et al. 2023). This retrospective study analyzed all consecutive cases of BMC diagnosed in breast cancer patients treated at two University Hospitals (Schleswig-Holstein Campus Lübeck and Düsseldorf) between January 1st 2010 and December 31st 2023. Inclusion criteria were as follows:

- Confirmed BMC diagnosis through bone marrow biopsy or leukoerythroblastic blood smear combined with cytopenia (Common Toxicity Criteria [CTC] grade ≥ 1).
- Availability of clinical data regarding tumor characteristics, hematological parameters, treatment regimens, and outcomes.

Data were extracted from patient records, including demographics (age, sex), tumor histology (e.g., ductal vs. lobular carcinoma), receptor status (ER/ PR/ HER2), prior treatments, and metastatic patterns. This analysis was carried out in accordance with the guidelines of the Declaration of Helsinki and approved by the Ethical Committee of the University of Lübeck (file number: 2024 − 440) and University of Düsseldorf (file number: 2024–2795).

### Literature review

To contextualize the findings, a literature review was conducted using PubMed with the search terms “Bone marrow carcinomatosis” OR “Bone marrow metastasis” AND “breast cancer” in accordance with the PRISMA guidelines (Page et al. 2021). Studies were included if they described five or more cases of bone marrow carcinomatosis in breast cancer patients. Key publications were reviewed to gain insights into clinical presentation, treatment responses, and survival outcomes in this rare metastatic manifestation (Figure S1).

### Statistical analysis

The data analysis was conducted using Excel 2503 and Statistical Package for Social Sciences (IBM SPSS Statistics, Version 29.0.2.0, Armonk, NY, USA: IBM Corp). Descriptive statistics were used to summarize patient characteristics and clinical outcomes. Kaplan-Meier survival analysis was performed to estimate overall survival (OS) from the time of BMC diagnosis. Subgroup analyses were conducted to explore potential prognostic factors and Kaplan-Meier graphs were conducted using GraphPad Prism, Version 10.5.0. Patient and tumor characteristics were described using appropriate summary statistics. Mean and standard deviation were calculated for continuous variables, while frequency and percentage were used for categorical variables. OS was defined as the time from the date of BMC diagnosis to death from any cause. Survival rates with 95% CI and median survival times were estimated using the Kaplan–Meier product limit method. Subgroups were displayed exploratively, and, due to the small subgroups, no statistical testing was performed.

## Results

### Estimated incidence of BMC

During the evaluation period from January 1st 2010 to December 31st 2023 a total of 7,651 patients with breast cancer were treated across the two study sites, with 2,794 patients (36.5%) at the Düsseldorf site and 4,857 patients (64.5%) at the Lübeck site. Among these, 844 patients in Lübeck (400 with primary and 444 with secondary metastatic disease) and 555 patients in Düsseldorf (202 with primary and 353 with secondary metastatic disease) had mBC. Overall, a BMC occurred in 2.6% of all patients with mBC and in 0.5% of all breast cancer patients irrespective of tumor stage (Figure S2).

### Patient characteristics

A total of 37 patients with BMC were included in the analysis. The mean age at BMC diagnosis was 56.2 years (CI 95%: 52.7, 59.8), with a median of 58.0 years (range 30–76 years). The majority of patients (86.4%) were older than 45 years. Most patients (70.3%) were postmenopausal at the time of primary breast cancer diagnosis.

At the time of BMC diagnosis, 89.2% of patients presented with HR positive and HER2 negative carcinomas, while 10.8% had HER2 positive disease. No triple-negative breast cancer (TNBC) cases were observed. Histologically, the predominant subtype was invasive carcinoma of no special type (59.5%), followed by invasive lobular carcinoma (35.1%). Tumor grading was most frequently G2 (62.2%).

More than half of the cohort (56.8%) had primary metastatic disease, while 43.2% developed secondary metastases. Bone marrow involvement was diagnosed mainly via biopsy (67.6%), and 59.5% of patients had visceral metastases at the time of BMC diagnosis (Table 1).

**Table 1:** Clinical characteristics of the patient cohort

Characteristic		All patients *N* = 37 (%)
Age (years)	Mean (SD and CI 95%)	56.2 (1.7; 52.7, 59.8)
	Median [range]	58 [30–76]
Metastatic site at time of BMC diagnosis	Bone only	12 (32.4)
	Bone + visceral	22 (59.5)
	Bone + CNS	1 (2.7)
	Bone + visceral + CNS	2 (5.4)
Timepoint of metastatic disease	Primary metastatic disease	21 (56.8)
	Secondary metastatic disease	16 (43.2)
Histological subtype primary disease	No special type	22 (59.5)
	Invasive lobular	13 (35.1)
	Other	1 (2.7)
	Unknown	1 (2.7)
Grading	G1	0 (0.0)
	G2	23 (62.2)
	G3	11 (29.7)
	Unknown	3 (8.1)
Menopausal status at the time of primary diagnosis	Pre-/perimenopausal	11 (29.7)
	Postmenopausal	26 (70.3)
Receptor profile	HR positive HER2 negative	33 (89.2)
	HER2 positive	4 (10.8)
	TNBC	0 (0.0)
End of observation	Last follow up	16 (43.2)
	Death	21 (56.8)
Time from first diagnosis of metastatic disease to BMC (in months)	Mean (SD; CI 95%)	32.0 (5.3; 21.3, 42.7)
	Median (range)	28 (0–107)
Time from first breast cancer diagnosis to BMC (in months)	Mean (SD; CI 95%)	62.3 (7.6; 46.8, 77.8)
	Median (range)	58 (0–249)
End of observation after BMC in months	< 13	22 (59.5)
	13 – 24	8 (21.6)
	25 – 36	4 (10.8)
	37 – 48	1 (2.7)

BMC: bone marrow carcinomatosis; CDK4/6- cyclin-dependent kinases 4/6; CI: conficende interval; CNS: central nervous system; HR: hormone receptor; HER2- human epidermal growth factor 2 receptor; TNBC: triple-negative breast cancer; SD: standard deviation

### Therapeutic approaches and clinical outcomes

Among patients with HR positive HER2 negative disease (*N* = 33), the most common systemic therapy received after BMC diagnosis was mono-chemotherapy (57.6%), followed by cyclin-dependent kinase 4/6 inhibitor- (CDK4/6i-) based treatment (18.2%) (Table 2). Most patients receiving mono-chemotherapy (63.2%) were treated with epirubicin weekly low-dose (30 mg/m^2^ i.v.). Other agents used in mono-chemotherapy were doxorubicin (20 mg/m^2^ i.v. weekly), paclitaxel (80 mg/m^2^ i.v. weekly), nab-paclitaxel (100 mg/m^2^ i.v. weekly), and capecitabine (2 × 500 mg p.o. daily fixed dose).

At BMC diagnosis, anemia of CTCAE grade ≥ 2 occurred in 72.9% of patients, thrombocytopenia of CTCAE grade ≥ 2 in 62.1%, and leukopenia of CTCAE grade ≥ 2 in 35.1%. Pancytopenia was observed in 56.8% of cases. Consequently, transfusion requirements were frequent: 62.1% of patients received 1–10 units of blood transfusions, with a median of 6.0 and a mean of 9.7 (CI 95%: 4.7, 14.8) transfusions per patient (Table 3).

The mean time from first breast cancer diagnosis to the development of BMC was 62.3 months (CI 95%: 46.8, 77.8; range 0–249; median 58.0), and from the onset of metastatic disease to BMC 32.0 months (CI 95%: 21.3, 42.7; range 0–107; median 28.0). In case of 10 patients (27.0%), BMC was the first manifestation of metastatic disease (4 patients with primary metastatic disease and 6 patients with secondary metastatic disease). At the end of follow-up, 56.8% of patients had died, while 43.2% had been lost to follow-up (Table 1).

**Table 2:** Prior therapies and primary treatment approaches in patients with BMC

Treatment characteristic		All patients *N* = 37 (%)
Number of treatments lines prior to BMC diagnosis (%) *	0	4 (10.8)
	1	2 (5.4)
	2	8 (21.6)
	3	5 (13.5)
	4	6 (16.2)
	5	5 (13.5)
	6	4 (10.8)
	7	1 (2.7)
	8	2 (5.4)
1st line therapy after BMC diagnosis and HR positive HER2 negative BC (*n* = 33)	Mono-chemotherapy	20 (60.6)
	Poly-chemotherapy or chemotherapy in combination with a VEGF antibody	1 (3.0)
	Chemotherapy in combination with a VEGF antibody	2 (6.1)
	CDK 4/6i-based therapy**	6 (18.2)
	Endocrine therapy only	1 (3.0)
	***Other	3 (9.1)
1st line therapy with BMC diagnosis and HER2 pos BC (*n* = 4)	Mono-chemotherapy with anti-HER2 therapy	1 (25.0)
	Mono-chemotherapy	2 (50.0)
	Best supportive care	1 (25.0)

BMC: bone marrow carcinomatosis; CDK4/6i- cyclin-dependent kinases 4/6 inhibitor; CI: confidence interval; HR: hormone receptor; HER2: human epidermal growth factor 2 receptor; SD: standard deviation; VEGF: vascular endothelial growth factor

* The number of therapies includes treatment lines for both eBC and mBC.

** 1x ribociclib; 2x abemaciclib, 3x palbociclib

*** Other including: 1× best supportive care, 1× everolimus plus exemestane, 1× alpelisib plus fulvestrant

**Table 3:** Laboratory findings at the time of BMC diagnosis and transfusion data

Diagnostics/Laboratory findings	Grade/Number	All patients *N* = 37 (%)
Diagnostic procedure for BMC diagnosis	Bone marrow biopsy	26 (70.3)
	Blood smear	11 (29.7)
Anemia in CTCAE Grade at BMC diagnosis	0	6 (16.2)
	I	4 (10.8)
	II	10 (27.0)
	III	13 (35.1)
	IV	4 (10.8)
Leukopenia in CTCAE Grade at BMC diagnosis	0	12 (32.4)
	I	12 (32.4)
	II	6 (16.2)
	III	2 (5.4)
	IV	5 (13.5)
Thrombocytopenia in CTCAE Grade at BMC diagnosis	0	9 (24.3)
	I	5 (13.5)
	II	6 (16.2)
	III	9 (24.3)
	IV	8 (21.6)
Pancytopenia at time of BMC diagnosis	yes	21 (56.8)
	no	16 (43.2)
Total number of erythrocyte transfusions per patient	Mean (SD; 95%CI)	9.7 (2.5; 4.7, 14.8)
	Median (range)	6.0 (0–74)
	0	1 (2.7)
	1–10	23 (62.1)
	11–20	6 (16.2)
	21–30	1 (2.7)
	31–40	0 (0.0)
	41–50	0 (0.0)
	> 51	2 (5.4)
	Unknown	4 (10.8)
Total number of thrombocyte transfusions per patient	Mean (SD; 95%CI)	1.5 (0.8; 0.0, 3.1)
	Median (range)	6 (0–19)
	0	20 (54.1)
	1–5	5 (13.5)
	6–10	3 (8.1)
	11–15	1 (2.7)
	16–20	1 (2.7)
	Unknown	7 (18.9)

BMC: bone marrow carcinomatosis; CTCAE: common terminology criteria for adverse events; CI: confidence interval; SD: standard deviation

### Survival analysis

**Fig. 1:**
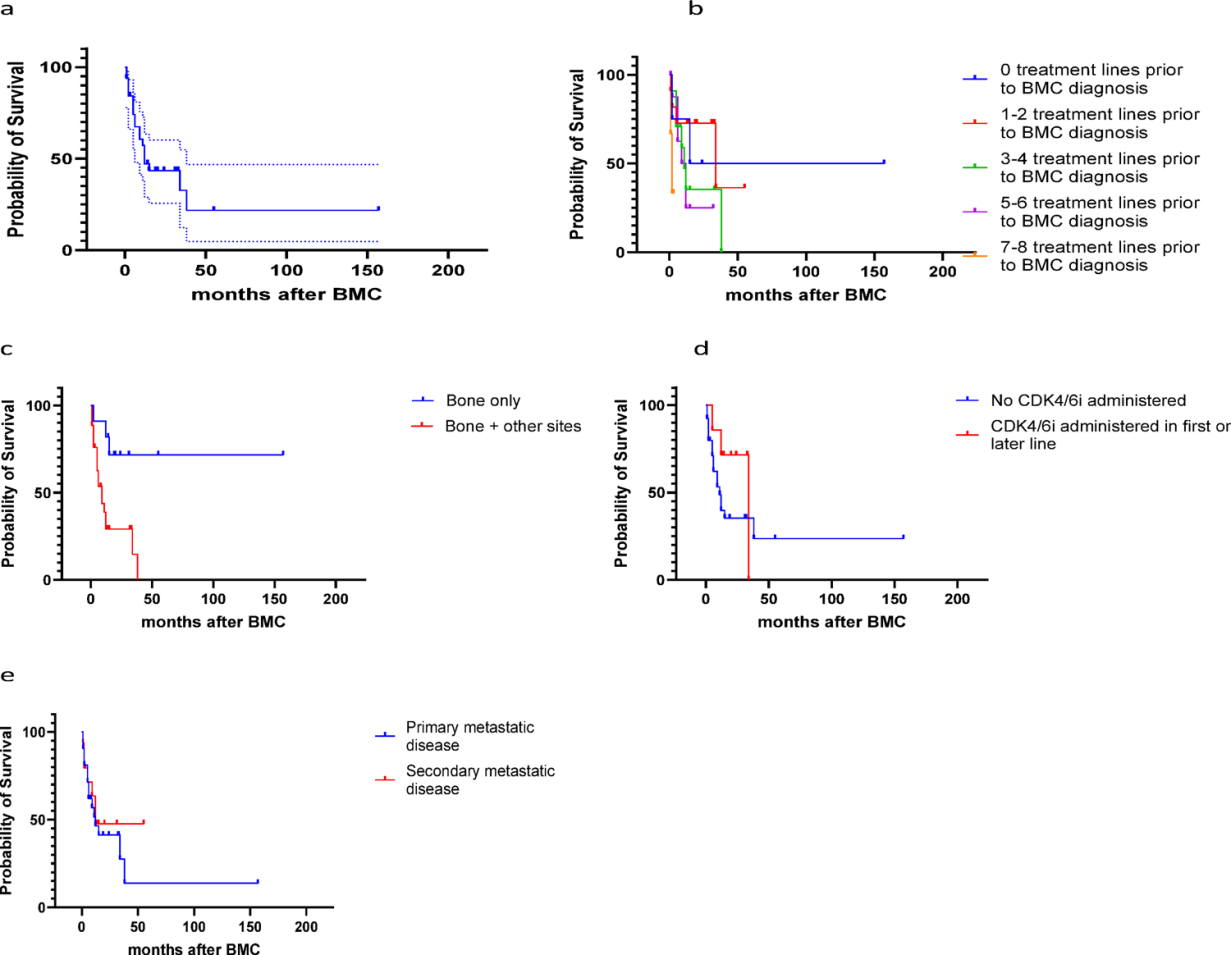
** a** Overall survival for all patients (*N* = 37).** b** Overall survival for patients according to their treatment lines prior to BMC diagnosis (0 prior treatment lines *N* = 4; 1–2 *N* = 10; 3–4 *N* = 11; 5–6 *N* = 9; 7–8 *N* = 3).** c** Overall survival after BMC diagnosis for all patients depending on the localization of metastatic sites (*N* = 37).** d** Overall survival depending on the use of CDK4/6i-based therapy after BMC diagnosis for patients with HR positive and HER2 negative mBC (*N* = 33).** e** Overall survival for all patients according to the time point of diagnosis of metastatic disease (*N* = 37).

At the time of analysis, 21 of 37 patients (56.8%) had died. The median OS after the diagnosis of BMC was 12.0 months (95% CI: 6.0, 38.0) (Fig. 1a; Table 4). Patients with 1–2 prior treatment lines before mBC diagnosis showed a median survival of 34.0 months (95% CI: 2.0, NA), compared with 11.0 months for patients with 3–4 prior lines and 10.5 months for those with 5–6 prior lines. Patients who had received 7–8 prior treatments had the shortest survival (2.0 months; CI 95%: 1.0, NA) (Fig. 1b; Table 4). Median survival was not reached in patients with bone-only disease, while those with additional visceral or other metastatic sites had a median survival of 9.0 months (95% CI: 5.0, 12.0) (Fig. 1c; Table 4).

Regarding first-line therapy after BMC diagnosis, patients treated with CDK4/6i demonstrated a numerically longer median survival (34.0 months; CI 95%: 5.0, NA) compared to those who received chemotherapy (12.0 months; 95% CI: 6.0, NA). Similarly, patients who received CDK4/6i therapy at any time after BMC diagnosis had a median survival of 34.0 months versus 11.0 months in those who did not (Fig. 1d; Table 4).

Median survival after BMC diagnosis did not differ substantially between patients with primary metastatic disease (12.0 months; CI 95%: 5.0, 38.0) and those with secondary metastatic disease (12.0 months; 95% CI: 2.0, NA) (Fig. 1e; Table 4).

**Table 4:** Survival data of the patient cohort

Outcome		*N*	Events	Median survival time in months (CI 95%)
All patients		37	21	12.0 (6.0, 38.0)
Age	Up to 45	6	2	NA
	46–60	18	8	15.0 (6.0, NA)
	60+	15	11	11.0 (5.0, NA)
Number of treatment lines prior to BMC diagnosis	1 or 2	15	6	34.0 (2.0, NA)
	3 or 4	11	7	11.0 (5.0, NA)
	5 or 6	9	6	10.5 (2.0, NA)
	7 or 8	3	2	2.0 (1.0, NA)
Metastatic site	OSS only	11	3	Median survival not reached
	OSS + Other	26	18	9.0 (5.0, 12.0)
1st line therapy with BMC and HR positive HER2 negative BC (*N* = 33)	Chemotherapy	27	17	12.0 (6.0, NA)
	CDK 4/6i	6	3	34.0 (5.0, NA)
CDK 4/6i therapy since BMC diagnosis with HR positive HER2 negative BC (*N* = 33)	Yes	7	3	34.0 (5.0, NA)
	No	26	16	11.0 (5.0, 38.0)
Timepoint of metastatic disease	Primary metastatic disease	21	14	12.0 (5.0, 38.0)
	Secondary metastatic disease	16	7	12.0 (2.0, NA)

BMC: bone marrow carcinomatosis; BC: breast cancer; CDK4/6i- cyclin-dependent kinases 4/6 inhibitor; CI: confidence interval; HR: hormone receptor; HER2: Human epidermal growth factor 2 receptor; NA: no data available; OSS: bone

## Discussion

To the best of our knowledge, this is the largest European cohort of breast cancer patients with bone marrow carcinomatosis reported to date. The observed prevalence of BMC is in line with previous studies. While epidemiological data on incidence of BMC in breast cancer patients are scarce, monocentric studies suggest that it occurs in approximately 0.17% among all BC cases (Kopp et al. 2011) and in 1.7–4.5% among those with advanced disease (Niu et al. 2023; Yang et al. 2022). Despite its rarity, the clinical implications are profound. Patients with BMC often present with bone metastases and unexplained cytopenias disproportionate to prior chemotherapy exposure. Importantly, no specific immunohistochemical breast cancer subtype or clinical characteristics have been definitively linked to an increased risk of BMC development to date, although most published cases have been reported in HR positive HER2 negative disease (Chavez-Macgregor et al. 2005; Demir et al. 2014; Kopp et al. 2011; Niu et al. 2023; Sakin et al. 2020; Yang et al. 2022). This underscores the need for heightened clinical vigilance in all patients with advanced disease.

Due to the limited evidence base, treatment recommendations lack strong scientific support and rely primarily on clinical experience and extrapolation from other metastatic settings. Current international guidelines, including those from the ESMO), NCCN, or ASCO, do not specifically provide detailed treatment recommendations for BMC in breast cancer. In contrast, the German Arbeitsgemeinschaft gynäkologische Onkologie (AGO) Breast guidelines recommend the use of weekly chemotherapy regimens- including epirubicin, doxorubicin, paclitaxel, and capecitabine, as well as anti-HER2 therapies for HER2 positive disease, while endocrine-based treatments for HR positive BMC receive only weak recommendations, all primarily based on small case series (Artac et al. 2014; Pahouja et al. 2015; Yamaguchi et al. 2020). In patients with BMC, epirubicin, alongside paclitaxel weekly, is frequently administered as a weekly low-dose regimen (30 mg/m² q1w), as it provides comparable efficacy with reduced myelotoxicity compared with standard q3w schedules. Weekly administration allows for sustained antitumor activity with improved hematologic tolerability, enabling stabilization or recovery of blood counts despite initial marrow infiltration Niu et al. 2023. Consequently, weekly regimens are generally preferred in patients with BMC.

When comparing the results of this analysis with previously published real-world data on BMC, the median OS of 12 months after diagnosis falls within the mid-range of reported outcomes (e.g., Kopp et al.: 19 months (Kopp et al. 2011); Niu et al.: 8 months (Niu et al. 2023); Sakin et al.: 9 months (Sakin et al. 2020); Yang et al.: 18 months (Yang et al. 2022) (Table 3). Differences may be attributed to patient selection factors, the observation period, and the use of modern therapeutic combinations. Notably, in our cohort, 54% of patients received monochemotherapy, 8% polychemotherapy, and 16% endocrine therapy (ET) combined with CDK4/6i- indicating a marked shift toward targeted therapies compared with older studies (e.g., Kopp et al., 0% ET + CDK4/6i (Kopp et al. 2011). Regarding molecular subtypes, HR positive HER2 negative tumors predominated (89%), followed by HER2 positive (11%), with no cases of TNBC observed. This trend is overall consistent with previously published real-world data (e.g., Niu et al., 73% (Niu et al. 2023); Yang et al., 76% (Yang et al. 2022), where HR positive subtypes also predominated, although the proportion of HR positive HER2 negative subtypes in our analysis was comparatively higher (Tables 3 and 5).

Niu et al. (2023) conducted a large single-center retrospective study in China (2010–2020) comprising 67 patients with BMC. ET led to the longest median survival (15.7 months), followed by polychemotherapy (9.7 months), with an estimated BMC prevalence of 2.1% among advanced breast cancers (Niu et al. 2023).

Yang et al. (2022) reported 25 out of 33 patients with HR positive HER2 negative mBC. A substantial proportion received ET (39%), while chemotherapy was administered in 55% of cases; however, median OS was markedly shorter in the chemotherapy group (5 months) compared to the entire cohort (18 months). Notably, ET was associated with significantly fewer adverse events, indicating superior tolerability in this patient population (Yang et al. 2022). Niu et al. (2023) and Yang et al. (2022) reported a relatively high proportion of patients treated with ET compared to those, receiving chemotherapy, representing a treatment distribution that stands in marked contrast to the therapeutic approach observed in many other cohorts (Demir et al. 2014; Kopp et al. 2011; Sakin et al. 2020). Among the 33 patients with HR positive HER2 negative mBC in our analysis, 7 received ET, of whom 6 were treated with a combination of ET and a CDK4/6i first-line therapy after BMC diagnosis. Patients treated with CDK4/6i also showed a markedly longer survival rates compared to those receiving chemotherapy alone (34 vs. 12 months), although these differences should be considered exploratory due to the small sample size.

Interestingly, 57% of the patients in this analysis presented with de novo metastatic breast cancer. This proportion is notably higher compared with the reference studies (Yang et al., 42% (Yang et al. 2022); Kopp et al., 32% (Kopp et al. 2011) (Table 5). The proportion of patients with isolated bone metastasis (bone only; 30%) has so far been reported in only one other study, in which Kopp et al. observed a higher rate of 45% (Kopp et al. 2011). At the same time, the median OS in that study was 19 months–7 months longer than in our cohort- which could be explained by the lower proportion of patients with visceral metastases.

**Table 5:** Literature review of published data about bone marrow carcinomatosis

Study	No of patients	Age (range/SD)	Histology	IHC subtype *n* (%)^1^	Metastatic status at initial BC presentation *n* (%)	Metastatic site at BMC diagnosis *n* (%)	Diagnostic method *n* (%)	Median time to BMC in months (range)	Clinical Symptoms *n* (%)	Median OS after BMC diagnosis in months (95% CI)	Initial treatment for BMC *n* (%)	Estimated incidence %
			a) IDC b) ILC c) unknown / other	a) HR positive HER2 negative b) HER2 positive c) TNBC	a) M0 b) M1	a) bone + VISC b) bone + CNS c) bone +/- other d) bone only e) bone + CNS + visceral	a) BM biopsy / smear b) blood smear	a) after mBC diagnosis b) after initial BC diagnosis	a) anemia b) thrombo-cytopenia c) leukopenia d) pan-cytopenia e) fever		a) combined CT b) mono CT c) ET+CDK4/6i d) ET only e) AntiHER2 f) BSC	
Kopp et al. single center, retrospective cohort study, 1995–2008 Germany (Kopp et al. 2011)	22	Median 47 (32–67)	a) 14 (64) b) 7 (32) c) 1 (4)	a) 12 (54)^2^ b) 3 (14)^2^ c) 2 (9) ^2^	a) 15 (68) b) 7 (32)	a) 10 (45) b) 0 (0) c) 22 (100) d) 10 (45) e) 2 (10)	a) 17 (77) b) 5 (23)	a) 31 (NA) b) 46 (2-196)	a) 17 (77) b) 9 (41) c) 5 (23) d) 1 (4) e) NA	19 (10.5–27.5)	a) 16 (73) b) 4 (18) c) 0 (0) d) 0 (0) e) NA f) 2 (9)	0.17^3^
Demir et al. single center,retrospective case-control study, 2003–2012 Turkey Demir et al. 2014)	27	Median 52 (30–77)	a) 19 (70) b) 7 (26) c) 1 (4)	a) 19 (70) b) 3 (11) c) 5 (19)	a) 13 (48) b) 14 (52)	a) NA) b) NA c) 27 (100) d) NA e) NA	a) 27 (100)	a) 6,5 b) 36.1 (1.6–70.5)	a) 22 (81) b) 11 (41) c) 7 (26) d) 4 (15) e) NA	6.43 (0–19.7)	a) 0 (0) b) 13 (52) c) 0 (0) d) 1 (4) e) NA f) 13 (44)	NA
Chavez-MacGregor et al. single center, retrospective cohort study, 1998–2001 Mexico (Chavez-Macgregor et al. 2005)	19	Mean 39.41 ( ± 12.07)	a) 17 (89) b) 2 (11)	NA	a) 8 (42) b) 11 (58	NA	a) 19 (100)	NA	NA	NA	NA	NA
Yang et al. single Center, retrospective cohort study, 2018–2022 China (Yang et al. 2022)	33	Median 50 (29–68)	a) 28 (85) b) 2 (6) c) 3 (9)	a) 25 (76) b) 3 (9) c) 5 (15)	a) 19 (58) b) 14 (42)	a) NA b) NA c) 33 (100) d) NA e) NA	a) 33 (100)	NA	a) 33 (100) b) 3 (9) c) NA d) 2 (6) e) 25 (76)	18 (2–18)	a)/b) 18 (55) c) 9 (27) d) 4 (12) e) 2 (6) f) 0 (0)	4.5^4^
Sakin et al. bicentric, retrospective cohort study, 2007–2017 Turkey (Sakin et al. 2020)	30	Median 45 (26–75)	a) 27(90) b) 3 (10)	a) 21 (70) b) 4 (13) c) 4 (13)	a) 16 (53) b) 14 (47)	NA	a) 30 (100%)	NA	a) 30 (100) b) 6 (20) c) NA d) 24 (80) e) NA	9 (3.63–14.36)	a) 0 (0) b) 27 (90) c) 0 (0) d) 0 (0) e) NA f) 3 (10)	NA
Niu et al. single center, retrospective cohort study, 2010–2020 China (Niu et al. 2023)	67	Median 48 (22–75)	NA	a) 49 (73) b) 9 (13) c) 9 (13)	a) 42 (63) b) 25 (37)	a) 44 (66) b) NA c) 67 (100) d) NA e) NA	a) 67 (100)	a) NA b) 52.1 (0-218.4)	a) 56 (84) b) 37 (55) c) 24 (36) d) 12 (18) e) 12 (18)	7.6 (3.9–11.3) ET: 15.7 vs. Combined CT: 9.7 vs. Mono CT: 8.6 vs. Untreated: 2.9	a) 13 (19) b) 17 (25) c) 3 (4) d) 18 (27) e) 2 (3) f)16 (24)	1.7^4^
Our study	37	Median 58 (30–76)	a) 22 (60) b) 13 (35) c) 2 (5)	a) 33 (89) b) 4 (11) c) 0 (0)	a) 16 (43) b) 21 (57)	a) 22 (60) b) 1 (3) c) 37 (100) d) 12 (32) e) 2 (6)	a) 26 (70) b) 11 (30)	a) 28 (0-107) b) 58 (0-249)	a) 31 (84) b) 24 (76) c) 25 (68) d) 21 (57) e) NA	12.0 (6.0, 38.0)	a) 3 (8) b) 20 (54) c) 6 (16) d) 1 (3) e) 1 (3) f) 1 (3) Other: 5 (14)	2.6^4^ 0.5^3^
BM: bone marrow; BMC: bone marrow carcinomatosis; BSC: best supportive care; CDK4/6i: cyclin-dependent kinases 4/6 inhibitor; CNS: central nervous system; CT: chemotherapy; ER: estrogen receptor; ET: endocrine therapy; HER2: human epidermal growth factor receptor 2; IDC: invasive ductal carcinoma; IHC: immunohistochemistry; ILC: invasive lobular carcinoma; NA: no data available; PR: progesterone receptor; SD: standard deviation; TNBC: triple-negative breast cancer; VISC: visceral metastasis. ^1^Percentages may not total 100 due to rounding or missing data; ^2^HER2 status was not determined in five patients treated before the approval of trastuzumab; ^3^of all initial breast cancer presentations; ^4^of advanced breast cancer cases,												

In more than 84% of patients, BMC manifested with anemia (at least CTCAE grade 1), in 76% with thrombocytopenia (at least CTCAE grade 1), and in 68% with leukopenia (at least CTCAE grade 1). Consistent with Kopp et al. (2011), Niu et al. (2023) and Demir et al. (2014) anemia was the most common hematological abnormality observed in our cohort, followed by thrombocytopenia and leukopenia (Demir et al. 2014; Kopp et al. 2011; Niu et al. 2023) (Table 5). The reported prevalence of pancytopenia varies considerably across studies, ranging from 4% (Kopp et al. 2011) to 80% (Sakin et al. 2020). In our analysis, the rate of pancytopenia was 57%. These findings underscore the need for heightened clinical suspicion of BMC in patients presenting with unexplained cytopenia and bone metastases (Kopp et al. 2011).

An additional aspect that should be considered when interpreting the incidence of BMC is the possibility of underdiagnosis. In heavily pretreated patients with mBC, cytopenias are frequently attributed to treatment-related bone marrow toxicity, particularly after multiple lines of chemotherapy or radiotherapy. However, persistent or disproportionate anemia, thrombocytopenia, leukopenia, or pancytopenia should raise clinical suspicion for bone marrow involvement, especially in patients with known bone metastases or otherwise unexplained hematologic abnormalities. Consequently, the true prevalence of BMC may be higher than reflected by retrospective cohorts relying on clinically established diagnoses. Early involvement of hematologic expertise may facilitate diagnostic work-up and help distinguish BMC from other therapy-associated bone marrow disorders, including treatment-related myelodysplastic syndrome (t-MDS) or malignancy associated hemophagocytic lymphohistiocytosis syndrome (HLH). In many cases, cytological assessment of bone marrow aspirates can already provide relevant diagnostic information and may allow differentiation between metastatic carcinoma infiltration and t-MDS before final histological results become available. This may support earlier treatment decisions and prevent misclassification of BMC-related cytopenias as treatment toxicity alone.

The findings of this study are consistent with previous research highlighting the rarity yet clinical importance of diagnosing BMC in metastatic breast cancer (Haborets and Shevnia 2025). They also underscore that the use of CDK4/6i instead of chemotherapy as first-line therapy for HR positive HER2 negative mBC following a BMC diagnosis is gaining increasing importance.

In interpreting prevalence data, it is important to note that most studies based their diagnoses exclusively on bone marrow biopsies (Chavez-Macgregor et al. 2005; Demir et al. 2014; Niu et al. 2023; Sakin et al. 2020; Yang et al. 2022), whereas Kopp et al. and our study also considered the presence of cytopenias as part of the diagnostic criteria for BMC; theoretically, this broader definition should yield higher prevalence rates in our cohort- yet this was not observed, raising questions about potential diagnostic underreporting or differing referral patterns.

With regard to therapeutic strategies, Niu et al. emphasized the limited evidence base for BMC treatment and highlighted that ET was associated with improved survival outcomes and better tolerability compared to chemotherapy (Niu et al. 2023). It is well recognized that CDK4/6i differ in their pharmacological properties, side effects and mechanisms of action, which must be considered when interpreting clinical outcomes. In the study by Niu et al. (2023), the intravenously administered CDK4/6i trilaciclib was used- an agent not available in our German cohort.

While other studies have shown that trilaciclib is effective in combination with chemotherapy- including in patients with metastatic TNBC- indicating a partially HR independent cytostatic effect (Tan et al. 2019), current ESMO, NCCN, and ASCO guidelines recommend the use of CDK4/6i in combination with ET and without additional chemotherapy as first-line treatment only for HR positive HER2 negative mBC (Al Sukhun et al. 2024; Gennari et al. 2021; Goetz et al. 2024; Hortobagyi Gabriel et al. 2022; Johnston et al. 2019, 2021; Lu et al. 2022; Rugo et al. 2019; Slamon et al. 2024; Tripathy et al. 2018). Independent of BMC, there is an ongoing discussion as to whether, in postmenopausal women with HR positive HER2 negative mBC, ET alone is sufficient as first-line treatment until disease progression, followed by a switch to CDK4/6i therapy as second-line treatment (Sonke et al. 2024). The recent publication of the RIGHT Choice study demonstrated that CDK4/6i can be effectively used even in the context of visceral crisis, challenging previous guideline recommendations that favored chemotherapy in such scenarios; a shift in clinical practice is expected following these findings in 2024 (Lu et al. 2024). While the RIGHT choice trial supports the use of CDK4/6i in the setting of visceral crisis, it does not specifically address BMC; however, data from Yang et al. and our analysis suggest that isolated BMC may be associated with favorable outcomes, particularly under ET and/or CDK4/6i (Lu et al. 2024; Yang et al. 2022). However, it should also be noted that CDK4/6i can cause significant neutropenia- usually reversibly. The molecular effects in the context of pre-existing BMC remain unclear. Furthermore, due to the small number of patients treated with CDK4/6i, it is not possible to draw correlations to determine whether this cohort exhibited lower bone marrow toxicity (e.g., lower rates of pancytopenia), which in turn could explain the significantly higher overall survival data compared to patients who did not receive CDK4/6i. This question needs to be addressed and discussed in larger studies.

### Strengths and limitations

The internal validity of this retrospective real-world analysis is supported by standardized data collection at two certified university breast cancer centers. The use of routinely documented clinical data minimizes the risk of recall bias, as no patient-reported or memory-based data were collected.

A potential selection bias is considered low, as in Germany, breast cancer patients represent the largest group of oncology patients treated in certified breast cancer centers (Schmitt et al. 2023). Moreover, the inclusion of patients from two independent university hospitals increases the heterogeneity of the study population and enhances generalizability. Therefore, the risk of limited representativeness is minimized, and external validity is deemed adequate for comparable certified clinical settings.

Due to the retrospective design, there is a risk of information bias, particularly through incomplete, misclassified, or inconsistently documented data. These potential sources of bias were addressed through standardized evaluation procedures to minimize their impact.

While leucoerythroblastic blood smears serve as a useful diagnostic tool, their sensitivity and specificity are suboptimal compared with bone marrow biopsy. Additionally, no specific tumor subtypes or molecular markers have been identified that reliably predict the risk of developing BMC; however, most patients in the published studies present with HR positive HER2 negative disease. Future research should aim to address these gaps by exploring biomarkers that could guide early diagnosis and personalized treatment strategies for this rare complication.

Most of the published literature consists of individual case reports or small case series, which provide valuable insights but lack statistical power and may be subject to publication bias. The absence of randomized controlled trials (RCT) specifically addressing BMC in breast cancer means that optimal treatment strategies have not been definitively established. Many recommendations are based on experience with BMC in other cancer types or general principles of managing metastatic breast cancer.

Given these limitations, current guidelines should be interpreted cautiously, and treatment decisions should be individualized based on patient characteristics, disease burden, and expert opinion.

However, being the largest cohort analyzed in the European setting to date, the total number of cases in our analysis (*N* = 37) remains low, which reflects the rarity of BMC. Statistical correlations within the entire cohort- for example, using a two-sided chi-squared test- could not be applied, as subgroup sizes were too small to get statistically valid results. Therefore, differences between subgroups should be interpreted as exploratory. Future statistical correlations could only be established and validated through larger registry databases. There is a clear need for larger, multi-center studies to better characterize the clinical course, prognostic factors, and treatment outcomes in BMC patients. Future efforts should focus on developing registries or collaborative networks to pool data on this rare condition and improve the evidence base for clinical decision-making.

## Conclusions

This bicentric retrospective analysis represents the largest European real-world cohort of patients with BMC. BMC remains a rare but clinically important manifestation, most commonly observed in HR positive HER2 negative tumors. The median OS (12 months) aligns with mid-range values from previous studies.

A key finding is the increasing use of ET combined with CDK4/6i, which appears to be associated with higher median survival rates compared to chemotherapy alone (34 vs. 12 months). However, this observation should be interpreted with caution, as the difference in survival may at least partly reflect a healthier patient cohort rather than a treatment effect.

Despite being the largest European cohort, the limited sample size restricts statistical power, and findings should be considered exploratory. Future multicenter registries and prospective studies are essential to refine diagnostic criteria, identify predictive biomarkers, and define optimal treatment strategies.

## Supplementary Information

Below is the link to the electronic supplementary material.

Supplementary Material 1

## Data Availability

No datasets were generated or analysed during the current study.
