# Supplementary material for: Real-world insights into bone marrow carcinomatosis in metastatic breast cancer: a retrospective analysis from two large university breast cancer centers
Source: J Cancer Res Clin Oncol. 2026 Jul 23;152(7):148. doi: 10.1007/s00432-026-06546-1 (PMC13396304; doi:10.1007/s00432-026-06546-1)
Supplement: Supplementary file 1 — Supplementary Material 1 [file 432_2026_6546_MOESM1_ESM.docx]

# Supplementary material

**Tauber et al.: Real-world insights into bone marrow carcinomatosis in metastatic breast cancer: a retrospective analysis from two large University Breast Cancer Centers, Journal of cancer research and clinical oncology**

Affiliation: University of Luebeck, Department of Gynecology and Obstetrics, University Hospital Schleswig-Holstein Campus Luebeck, 23538 Lübeck, Germany; Nikolas.Tauber@uksh.de

Figure S1: Literature collection in accordance with the PRISMA guideline


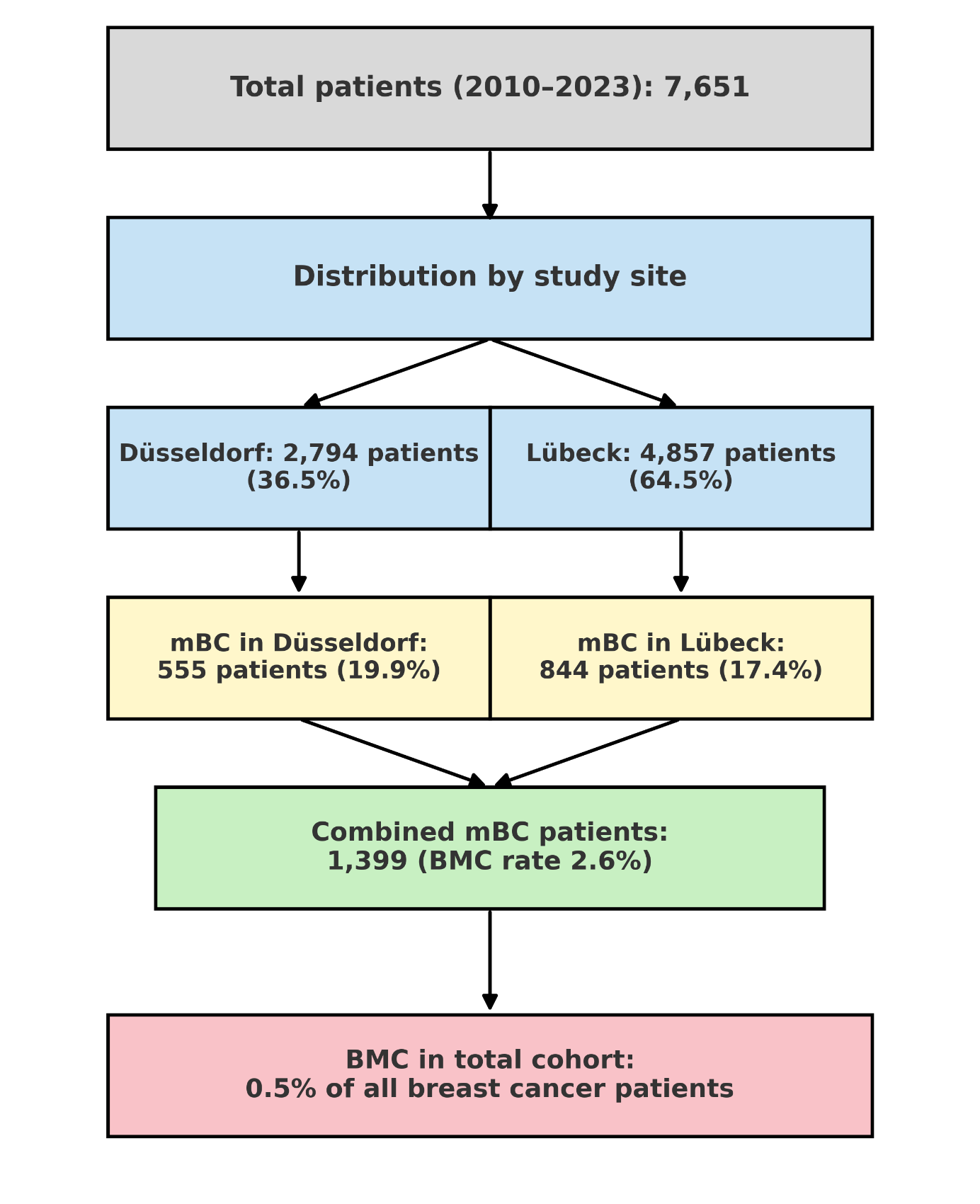


Figure S2: Distribution of breast cancer patients treated at the Düsseldorf and Lübeck study sites (2010–2023) and corresponding rates of mBC and BMC
